# Performance of GPT-3.5 and GPT-4 on the Japanese Medical Licensing Examination: Comparison Study

**DOI:** 10.2196/48002

**Published:** 2023-06-29

**Authors:** Soshi Takagi, Takashi Watari, Ayano Erabi, Kota Sakaguchi

**Affiliations:** 1 Faculty of Medicine Shimane University Izumo Japan; 2 General Medicine Center Shimane University Hospital Izumo Japan; 3 Department of Internal Medicine University of Michigan Medical School Ann Arbor, MI United States; 4 Medicine Service VA Ann Arbor Healthcare System Ann Arbor, MI United States

**Keywords:** ChatGPT, Chat Generative Pre-trained Transformer, GPT-4, Generative Pre-trained Transformer 4, artificial intelligence, AI, medical education, Japanese Medical Licensing Examination, medical licensing, clinical support, learning model

## Abstract

**Background:**

The competence of ChatGPT (Chat Generative Pre-Trained Transformer) in non-English languages is not well studied.

**Objective:**

This study compared the performances of GPT-3.5 (Generative Pre-trained Transformer) and GPT-4 on the Japanese Medical Licensing Examination (JMLE) to evaluate the reliability of these models for clinical reasoning and medical knowledge in non-English languages.

**Methods:**

This study used the default mode of ChatGPT, which is based on GPT-3.5; the GPT-4 model of ChatGPT Plus; and the 117th JMLE in 2023. A total of 254 questions were included in the final analysis, which were categorized into 3 types, namely general, clinical, and clinical sentence questions.

**Results:**

The results indicated that GPT-4 outperformed GPT-3.5 in terms of accuracy, particularly for general, clinical, and clinical sentence questions. GPT-4 also performed better on difficult questions and specific disease questions. Furthermore, GPT-4 achieved the passing criteria for the JMLE, indicating its reliability for clinical reasoning and medical knowledge in non-English languages.

**Conclusions:**

GPT-4 could become a valuable tool for medical education and clinical support in non–English-speaking regions, such as Japan.

## Introduction

ChatGPT (Chat Generative Pre-trained Transformer; OpenAI) is a state-of-the-art large language model (LLM) that can simulate human-like conversations based on user input [1]. As a continually evolving model in natural language processing (NLP), ChatGPT has the potential to be a valuable tool for clinical support and medical education, as already explored by Microsoft and OpenAI [2]. Studies have revealed that ChatGPT provided highly accurate answers to the US Certified Public Accountant exam and the US bar exam [3,4]. In the medical domain, ChatGPT achieved the passing criteria for the US Medical Licensing Examination (USMLE) [5,6]. Although challenges persist in applying ChatGPT to clinical medicine [7-9], it has demonstrated sufficient performance in English examinations [10].

However, in a previous study, ChatGPT, based on GPT-3.5 (Generative Pre-trained Transformer), performed poorly for 77 out of 79 medical students on a South Korean parasitology examination, which resulted in questions about its ability to provide medically accurate responses in non-English languages [11]. On March 14, 2023, OpenAI unveiled GPT-4, the latest version of its LLM [12]. Compared with its predecessor GPT-3.5, GPT-4 is “more reliable, creative, and able to handle many more nuanced instructions” [12]. OpenAI announced that GPT-4 could perform well in academic and specialized fields [12,13], and its performance in languages other than English was enhanced. However, OpenAI has yet to verify the performance of GPT-4 in the medical field in Japanese. When considering the application of GPT-4 to medical education and clinical practice in non–English-speaking regions, confirming its reliability for clinical reasoning and medical knowledge in non-English languages is critical [14].

Therefore, this study compared the accuracy of GPT-3.5 and GPT-4 on the Japanese Medical Licensing Examination (JMLE) [15]. Furthermore, the accuracy of each model was compared for various question types and difficulty levels.

## Methods

### Overview

We used the default mode of ChatGPT, which is based on GPT-3.5, and the GPT-4 model of ChatGPT Plus. The latest JMLE, number 117, conducted on February 4 and 5, 2023, was also used for this study. The JMLE comprises 400 questions, which were classified into 3 categories: essential knowledge questions, which test the knowledge and ethics required of a doctor; general clinical questions, which cover numerous diseases; and specific disease questions, which test the knowledge of each disease [15]. Furthermore, we categorized those questions into 3 types: general questions that tested knowledge of a specific topic, clinical questions that required case presentation and clinical reasoning, and clinical sentence questions with several questions in a single case. The passing criteria of the 117th JMLE are as follows: a minimum score of 80% on the essential knowledge questions and 74.6% on the remaining questions [15,16]. The exclusion criteria included questions for which the Ministry of Health, Labour and Welfare (MHLW) announced as being excluded (n=5), as well as questions containing tables (n=7), images (n=125), and underlining (n=9), which are not recognized by ChatGPT. In total, 254 questions were used in the final analysis.

Questions and their multiple-choice answers from the JMLE were used in their original Japanese form, as was the official national examination rubric. Instructions for using ChatGPT were also provided in Japanese. A typical rubric is as follows:

We will present questions for the Japanese National Medical Examination. There will be five options from a to e, and you must choose the appropriate option for the question. If there is no specific limit on the number of options to choose, please select one option only.

15

The definition of “correct” answers to the questions asked to GPT-3.5 and GPT-4 was based on the answers to the JMLE, which were published on the website of the MHLW [15]. Only the answers that were clearly correct and followed the instructions provided in the question text were considered “correct.” Ambiguous answers, evident mistakes, and responses with an excessive number of candidates were considered incorrect.

We evaluated the difficulty level of each question and categorized them as hard (n=82), normal (n=112), and easy (n=60) based on the correct response rate published by medu4, a preparatory school for the JMLE [16,17]. Questions with a correct response rate of 79.9% or below were classified as hard, those with a rate between 80% and 96.9% were classified as normal, and those with a rate of 97% or higher were classified as easy.

Finally, we simultaneously collected responses from both GPT-3.5 and GPT-4 between March 16 and 18, 2023, and scored them using the definition of correct answers. Multimedia Appendix 1 shows examples of the JMLE questions inputted into both models.

Standard descriptive statistics were used to calculate the numbers, proportions, and means for each data set. The McNemar test was used to compare correct response rates. All analyses were performed using the Stata statistical software (StataCorp LLC) [18]. All tests were 2-tailed, and statistical significance was set at *P*<.05.

### Ethical Considerations

This study only used information that was already published on the internet and did not involve human subjects; rather, an analysis of the JMLE was performed. Therefore, approval by the Institutional Review Board of Shimane University was not required.

## Results

A total of 254 questions from the 117th JMLE were used in the experiment. Table 1 presents the percentage of correct responses to essential knowledge questions and other questions on the JMLE. Overall, GPT-4 significantly outperformed GPT-3.5 by 29.1% (*P*<.001). In terms of the correct response rate for individual questions, the examinees’ rate for essential knowledge questions was 89.2% compared to 87.2% for GPT-4. Notably, this represents a considerable 32.1% improvement over GPT-3.5, which had a 55.1% correct response rate. Similarly, a 29.5% increase was observed for general clinical questions, and a 25.4% increase was observed for specific disease questions. In all cases, GPT-4 achieved the passing rates for the JMLE. However, none of these rates exceeded the total percentage of correct answers by examinees.

Table 2 presents the correct response rates according to the question type, with GPT-3.5 achieving correct response rates of approximately 50%—none of which are passing scores. However, GPT-4 achieved a 27.6% increase for general questions (*P*<.001) and a 29.6% increase for clinical questions (*P*<.001) compared to GPT-3.5. Notably, a 36.3% increase was observed in the number of correct responses to clinical sentence questions, with a significant improvement in all question types (all *P*<.05).

Table 3 presents the correct response rates by difficulty level. GPT-3.5 only achieved a 69.5% correct response rate for easy-level questions, 46.2% for normal-level questions, and 33.3% for hard-level questions. None of these values were close to the passing criteria. However, GPT-4 exhibited improved performance, with a 40% increase for hard-level questions (*P*<.001), a 31.5% increase for normal-level questions (*P*<.001), and an 18.3% increase for easy-level questions (*P*<.001).

Finally, GPT-4 significantly outperformed GPT-3.5 in all formats in terms of correct response rates (all *P*<.05). In particular, for hard-level questions, the correct response rate of GPT-4 was 17% higher than the examinees’ average correct response rate.

**Table 1:** Comparison of GPT-3.5 (Generative Pre-trained Transformer) and GPT-4 for essential knowledge questions and other questions in the Japanese Medical Licensing Examination (JMLE).

Question category	Question (n=254), n (%)	Examinee correct response rate^a^ (%)	GPT-3.5 correct response rate (%; 95% CI)	GPT-4 correct response rate (%; 95% CI)	*P* value
All questions	254 (100)	84.9	50.8 (44.6-57.0)	79.9 (75.0-84.9)	<.001
Essential knowledge	78 (30.7)	89.2	55.1 (43.8-66.4)	87.2 (79.6-94.8)	<.001
General clinical	105 (41.3)	83.1	43.8 (34.2-53.5)	73.3 (64.7-81.9)	<.001
Specific disease	71 (28)	83	56.3 (44.5-68.2)	81.7 (72.5-90.9)	<.001

^a^The correct response rates of examinees were obtained from the 117th JMLE, as announced by the Ministry of Health, Labour and Welfare [15].

**Table 2:** Comparison of GPT-3.5 (Generative Pre-trained Transformer) and GPT-4 by question type in the Japanese Medical Licensing Examination (JMLE).

Question type	Question (n=254), n (%)	Examinee correct response rate^a^ (%)	GPT-3.5 correct response rate (%; 95% CI)	GPT-4 correct response rate (%; 95% CI)	*P* value
General	134 (52.7)	84	51.5 (42.9-60.0)	79.1 (72.1-86.1)	<.001
Clinical	98 (38.6)	85.3	50 (39.9-60.1)	79.6 (71.5-87.7)	<.001
Clinical sentence	22 (8.7)	88.8	50 (27.3-72.7)	86.3 (70.8-102)	.005

^a^The correct response rates of examinees were obtained from the 117th JMLE, as announced by the Ministry of Health, Labour and Welfare [15].

**Table 3:** Comparison of GPT-3.5 (Generative Pre-trained Transformer) and GPT-4 in the Japanese Medical Licensing Examination (JMLE) by difficulty levela.

Difficulty level	Question (n=254), n (%)	Examinee correct response rate^b^ (%)	GPT-3.5 correct response rate (%; 95% CI)	GPT-4 correct response rate (%; 95% CI)	*P* value
Easy	82 (32.3)	98.7	69.5 (59.3-79.7)	87.8 (80.6-95.0)	.001
Normal	112 (44.1)	90.2	46.2 (37.0-55.8)	77.7 (69.8-85.5)	<.001
Hard	60 (23.6)	56.3	33.3% (21.1-45.6)	73.3 (61.8-84.8)	<.001

^a^Difficulty level was classified by the percentage of correct responses provided by medu4 [16], Japan’s leading preparatory school for the JMLE: easy, >97%; normal, 80% to 96.9%; and hard, <79.9%.

^b^The correct response rates of examinees were obtained from the 117th JMLE, as announced by the Ministry of Health, Labour and Welfare [15].

## Discussion

### Principal Findings

We compared the correct response rates of GPT-3.5 and GPT-4 on the 2023 JMLE. GPT-3.5 did not satisfy the passing criteria, whereas GPT-4 achieved the required scores. Furthermore, GPT-4 demonstrated a significantly improved correct response rates compared with GPT-3.5 across various question types and difficulty levels. The correct response rate of GPT-4 was particularly enhanced for the challenging hard-level questions and surpassed the average correct response rate of actual examinees. Based on these results, we discuss 2 factors that explain the significant improvement in the correct response rates of GPT-4 on the JMLE.

First, we ascribe this enhancement to the augmented NLP capabilities in non-English languages. A performance disparity between English and other languages in LLMs is ubiquitous in NLP [19]. Additionally, GPT-3.5 exhibits a decline in NLP proficiency in non-English languages relative to English [20]. Although GPT-3.5 passed the USMLE, an English language–based medical examination, it did not satisfy the passing criteria for the JMLE. In contrast, GPT-4 satisfied the JMLE passing criteria, demonstrating a significant advancement in NLP capabilities, specifically in Japanese. OpenAI assessed GPT-4’s performance in non-English languages, which yielded higher proficiencies in 24 out of 26 languages as compared to the previous models’ proficiency in English [13]. Although OpenAI did not disclose the precise methodologies used to obtain these outcomes, the results of this research validate their assertion.

Second, since improving the information processing capabilities in professional and academic domains is imperative, OpenAI’s development of GPT-4 aimed to handle more intricate and nuanced tasks beyond those encountered in many real-world situations [13]. The JMLE is a mandatory exam for certifying medical practitioners in Japan, necessitating a comprehensive knowledge base and strong clinical reasoning skills. GPT-3.5’s performance fell short of the JMLE passing criteria, whereas GPT-4 made significant improvements in professional and academic processing capabilities in a brief time frame. Notably, GPT-4’s superior correct response rate on the challenging hard-level questions, compared with the average correct response rate of general examinees, indicates the potential of language models such as GPT-4 to surpass human performance in highly specialized fields [13].

As the results of this study and several previous studies indicate, LLMs such as ChatGPT have made remarkable progress [2,7,13]. However, we should be careful when directly applying LLMs in clinical practice and education without critical scrutiny [9]. For example, the most essential challenge to address is hallucination. Hallucination is defined as “producing nonsensical or untruthful content concerning certain sources.” OpenAI reported that hallucinations have been mitigated in GPT-4 compared with GPT-3.5 [21]. With advancements in LLMs, hallucinations may be further reduced in the future. Future studies should discuss the quality level of LLMs that is required. A previous study suggests that even in English, in a real clinical setting, GPT-3.5 cannot answer questions at a level acceptable to fully qualified primary care physicians [10]. However, LLMs such as GPT-4 exhibit considerable potential for use in clinical sites and medical education. For instance, ChatGPT has been used to generate differential diagnoses [22]. Furthermore, the potential of ChatGPT for improving the diagnosis and treatment of epilepsy and contributions to public health improvement has been investigated [23-25].

### Limitation

This study had several limitations. First, the results reflect the capabilities of ChatGPT as of March 17 and 18, 2023, and different results could be obtained even if the same methods were used. The knowledge and interpretation capabilities of ChatGPT will rapidly improve in the future because of user feedback and deep learning. Second, although GPT-4 is a multimodal artificial intelligence that is inherently capable of inputting images and tables, among other things, this study excluded them for an accurate comparison with GPT-3.5, and only text questions were used. Third, the JMLE has a supplementary assessment that states that if an absolute contraindication answer is selected 2 or more times, the applicant will fail the examination, even if they have achieved the passing scores [15]. Because the scores of failed applicants were not published by the MHLW, they were not included in the evaluation. Finally, this investigation focused exclusively on ChatGPT. However, other LLMs such as Google’s Bard (PaLM2) and Large Language Model Meta AI (LLaMA) have advanced considerably and are being improved continuously [26]. In the future, the possibility of implementing LLMs other than ChatGPT in the medical field must be considered.

### Conclusions

GPT-4 passed the 117th JMLE, whereas GPT-3.5 failed the examination. This phenomenon revealed GPT-4’s rapid evolution in Japanese language processing. Investigations are necessary to evaluate its safety, efficiency, and cost-effectiveness for potential application as an LLM artificial intelligence tool for medical practice support, learning in clinical settings, and medical education.

## Appendix

Multimedia Appendix 1 Examples of the Japanese Medical Licensing Examination questions inputted into ChatGPT (Chat Generative Pre-trained Transformer; left) and GPT-4 (Generative Pre-trained Transformer-4; right). In the instructions, the text of the Japanese National Medical Examination was used as it is, without any changes.

## Abbreviations

ChatGPT: Chat Generative Pre-trained Transformer
GPT: Generative Pre-trained Transformer
JMLE: Japanese Medical Licensing Examination
LLaMA: Large Language Model Meta AI
LLM: large language model
MHLW: Ministry of Health, Labour and Welfare
NLP: natural language processing
USMLE: US Medical Licensing Examination

## Data Availability

Data supporting the findings of this study are available from the corresponding author (TW) upon request.
