# Supplementary figures and images for: Performance of GPT-3.5 and GPT-4 on the Japanese Medical Licensing Examination: Comparison Study
Source: JMIR Med Educ. 2023 Jun 29;9:e48002. doi: 10.2196/48002 (PMC10365615; doi:10.2196/48002)

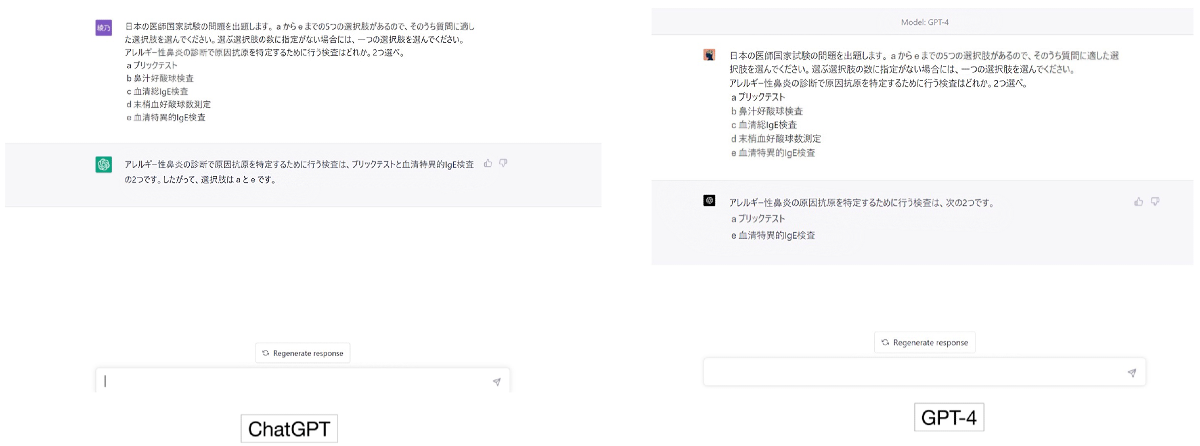

Supplement: Multimedia Appendix 1 [file mededu_v9i1e48002_app1.png]
